# Disorders of Sleep and Ventilatory Control in Prader-Willi Syndrome

**DOI:** 10.3390/diseases4030023

**Published:** 2016-07-08

**Authors:** Emily S. Gillett, Iris A. Perez

**Affiliations:** Division of Pediatric Pulmonology and Sleep Medicine, Children’s Hospital Los Angeles, 4650 Sunset Blvd, Mailstop #83, Los Angeles, CA 90027, USA; iaperez@chla.usc.edu

**Keywords:** Prader-Willi syndrome, obstructive sleep apnea, sleep disorders, ventilatory control, obesity, growth hormone, adenotonsillectomy, non-invasive ventilation, bariatric surgery, hypocretin

## Abstract

Prader-Willi syndrome (PWS) is an imprinted genetic disorder conferred by loss of paternal gene expression from chromosome 15q11.2-q13. Individuals with PWS have impairments in ventilatory control and are predisposed toward sleep disordered breathing due to a combination of characteristic craniofacial features, obesity, hypotonia, and hypothalamic dysfunction. Children with PWS progress from failure to thrive during infancy to hyperphagia and morbid obesity during later childhood and onward. Similarly, the phenotype of sleep disordered breathing in PWS patients also evolves over time from predominantly central sleep apnea in infants to obstructive sleep apnea (OSA) in older children. Behavioral difficulties are common and may make establishing effective therapy with continuous positive airway pressure (CPAP) more challenging when OSA persists after adenotonsillectomy. Excessive daytime sleepiness (EDS) is also common in patients with PWS and may continue after OSA is effectively treated. We describe here the characteristic ventilatory control deficits, sleep disordered breathing, and excessive daytime sleepiness seen in individuals with PWS. We review respiratory issues that may contribute to sudden death events in PWS patients during sleep and wakefulness. We also discuss therapeutic options for treating sleep disordered breathing including adenotonsillectomy, weight loss, and CPAP. Lastly, we discuss the benefits and safety considerations related to growth hormone therapy.

## 1. Introduction

Prader-Willi syndrome (PWS) is a genetic disorder estimated to affect between 1 in 10,000 and 1 in 30,000 live births [1,2,3]. PWS is caused by loss of imprinted gene expression from the paternal copy of chromosome 15q11.2-q13. This is most commonly caused by deletions in the paternal copy of chromosome 15 but can also be due to maternal uniparental disomy, paternal chromosomal translocation, or failures in DNA methylation essential to regulate gene expression [3,4,5,6]. Multiple genes in this region are thought to contribute to the PWS phenotype.

Consensus clinical criteria for diagnosis vary depending on the age of the child. In the prenatal and infant time periods, features include hypotonia, decreased fetal movement, infantile lethargy, and feeding problems [3,7]. After infancy, children begin to exhibit hyperphagia, and obesity is further exacerbated by a tendency toward short stature [3,7]. Hypogonadism and pubertal delay are typical, as are certain facial features such as almond-shaped eyes, narrow nasal bridge, thin upper lip and down-turned corners of the mouth [3,7]. Individuals with PWS rarely vomit and may have blunted responses to external heat or cold [3]. Hypothalamic dysfunction may lead to high pain threshold, temperature instability, and endocrine dysfunction including deficiencies in growth hormone or thyroid stimulating hormone, and central adrenal insufficiency [3,8]. Cognitive disability of varying severity is seen, along with characteristic behaviors such as temper tantrums, resistance to change, manipulative tactics, and compulsiveness [3,7]. Difficult behaviors overlap with those characteristically seen in children with autism spectrum disorder, and such behaviors may make instituting therapeutic interventions that require patient cooperation more challenging. Some psychological problems become worse as individuals age, and about 10% to 20% of young adults with PWS will develop psychosis [3]. Although mild to moderate cognitive disability is typical, it has also been noted that some people with PWS are especially skillful in solving visual-perceptual motor challenges, such as jigsaw puzzles [7].

Ventilatory control responses to hypoxemia and hypercapnia are altered in PWS. A high percentage of individuals with PWS exhibit sleep disordered breathing which may be severe [9,10]. Estimated mortality risk for individuals with PWS is increased at 3% per year across all age groups [11]. Cause of death is often linked to respiratory infection or respiratory disorder and may be sudden, with some reported cases of sudden death occurring at night [12]. It is therefore important to test for and treat sleep-related breathing disorders in this population to preserve cardiovascular health, improve daytime functioning, and limit the risk of severe respiratory events during sleep. These patients may be particularly vulnerable to severe respiratory events during acute respiratory illness and initiation of growth hormone (GH) treatment. In this paper, we describe the characteristic ventilatory control deficits, sleep disordered breathing, and excessive daytime sleepiness seen in individuals with PWS. We review respiratory issues that may contribute to sudden death events in PWS patients during sleep and wakefulness. We also discuss therapeutic options for treating sleep disordered breathing and considerations related to GH therapy.

## 2. Abnormalities of Ventilatory Control

### 2.1. Hypoxic Ventilatory Response (HPVR)

The hypoxic ventilatory response (HPVR) is mediated by peripheral chemoreceptors located in the carotid bodies that communicate with central respiratory control centers to modulate respiratory rate and the tidal volume of each breath. The HPVR increases minute ventilation in order to maintain blood oxygen levels in the normal range under hypoxic conditions (such as at high altitude) and following desaturations related to apneic pauses. Atmospheric air at sea level contains 21% O_2_. When asked to breathe air containing only 14% O_2_, 35% of subjects with PWS exhibited no HPVR response and the remainder of PWS subjects had a significantly blunted response compared to healthy controls [13]. In addition, PWS subjects who did mount a HPVR response tended to increase tidal volume of each breath more than respiratory rate, which was the opposite of healthy controls [13]. Paradoxical responses to hyperoxia have also been documented in subjects with PWS where breathing a higher concentration of O_2_ was found to increase, rather than decrease, minute ventilation [14]. Alterations in ventilatory control, such as an abnormal HPVR, are posited to play a role in the pathophysiology of sleep-disordered breathing in some individuals with PWS [13].

### 2.2. Hypercapnic Ventilatory Response (HCVR)

During prolonged apneas or periods of hypoventilation, blood oxygen levels fall while CO_2_ levels may rise. Elevations in the partial pressure of arterial CO_2_ are detected by peripheral chemoreceptors at the carotid bodies, while central chemoreceptors respond primarily to decreases in the pH of blood and cerebral spinal fluid associated with increased CO_2_ and respiratory acidosis. In contrast to HPVR, the hypercapnic ventilatory response (HCVR) measures an individual’s ventilatory response to elevated concentrations of CO_2_. When HCVR was tested in subjects with and without PWS, all individuals showed some increase in ventilation in response to hypercapnia. However, obese subjects with PWS had a blunted HCVR compared to non-obese subjects with PWS and BMI-matched obese controls [13]. Blunting of the HCVR may provide yet another impediment to obese individuals with PWS maintaining normoxia and normocapnia in an efficient manner. Interestingly, treatment with growth hormone (GH) for 6–9 months has been shown to improve resting ventilation and ventilatory response to CO_2_ in subjects with PWS in a manner that is not correlated with improvements in BMI [15]. The mechanism underlying this effect of GH on ventilation is unknown, but these results suggest that GH may play an important role in modulating the sensitivity of peripheral chemoreceptors to CO_2_, or in relaying input from peripheral chemoreceptors to central respiratory control centers. Animal studies have suggested that the posterior hypothalamus plays a role in the ventilatory response to CO_2_ [16], so GH could also be exerting its effects at that level. During sleep, GH therapy in children with PWS leads to a modest improvement in SaO_2_ during sleep but does not lead to significant changes in responsiveness to hyperoxia or hypercarbia, perhaps because the hypothalamus exerts less influence on respiratory drive during sleep [17].

## 3. Pulmonary Mechanics

The efficiency of the respiratory system in maintaining normal levels of oxygen and carbon dioxide in the blood is determined by the effectiveness of ventilatory control mechanisms and the mechanical properties of the respiratory system. Several physical traits associated with PWS contribute to suboptimal pulmonary mechanics including hypotonia, respiratory muscle weakness, scoliosis or kyphoscoliosis, and obesity.

### 3.1. Hypotonia and Respiratory Muscle Weakness

Hypotonia and decreased lean muscle mass are prominent features of PWS. These traits may lead to problems with pulmonary function through decreased respiratory muscle strength and decreased upper airway tone, particularly during sleep. Children and young adults with PWS exhibit a restrictive ventilatory impairment compared to normative ranges [18]. This is seen in both obese and non-obese individuals and is thought to be due, at least partly, to reduced ventilatory muscle strength. In general, restrictive ventilatory defects reduce tidal volume and therefore increase the respiratory rate and work of breathing necessary to maintain stable blood levels of oxygen and carbon dioxide, particularly during periods of physical stress or illness.

Restrictive ventilatory defects related to muscle weakness in PWS may be further exacerbated by scoliosis or kyphoscoliosis, as these spinal deformities alter chest wall mechanics impairing pulmonary function and increasing the likelihood of alveolar hypoventilation [19]. Expiratory respiratory muscle weakness may also lead to poor cough effectiveness and difficulty clearing airway secretions. In individuals with neuromuscular weakness, expiratory muscle strength and cough effectiveness are further reduced during acute upper respiratory tract infections when they are most essential to clear airway secretions [20]. During acute illness in PWS patients, hypothalamic dysfunction and possible defects in central adrenal regulation may lead to dysautonomia with lack of fever (or very high fever) and poor stress response [21]. Abnormal ventilatory control and difficulty with effective airway clearance may further increase the vulnerability of individuals with PWS during acute respiratory illnesses which are sometimes associated with sudden death in this population [22,23].

### 3.2. Obesity

Obesity is common in PWS, but the relationship between obesity and pulmonary function in children is complex. In adults, increasing obesity is associated with increased airway resistance [24]. Lung compliance in adults is also reduced [25,26], and lower lung volumes are seen due to the restrictive effects of excess weight on expansion of the chest wall and downward movement of the diaphragm toward the abdomen [26,27,28]. In children, the optimal range for body mass index (BMI) is dependent on age, and BMI percentage is not clearly correlated with specific changes in lung function. Some pediatric studies have shown that obese children, or those with increased percentage of total body fat, have a decrease in functional residual capacity (FRC) which is the resting volume of the lung at end expiration during normal tidal breathing [29,30,31], while other studies have not found a significant difference in lung volumes between obese and healthy weight children [32,33,34]. Some investigators have shown that longer duration of obesity is a predictor of lower FRC and worse pulmonary function [35], so it may be that obesity has a greater impact on the pulmonary function of adults with PWS. In children with PWS, alterations in pulmonary function may be more greatly affected by respiratory muscle weakness as well as additional pulmonary issues linked to obesity, such as asthma [36,37]. Obesity in children has also been shown to produce expiratory flow limitation during exercise, suggesting that obesity further perturbs ventilatory function in obese children when the cardiorespiratory system is under increased stress and demand [38].

## 4. Sleep Disordered Breathing

### 4.1. Obstructive Sleep Apnea (OSA)

The prevalence of OSA in children with PWS is just under 80% according to recent meta-analysis [9], which is much higher than the 1% to 4% prevalence seen in the general pediatric population [39]. Altered ventilatory control, obesity, airway hypotonia, micrognathia, narrowing of the upper airway, and respiratory muscle weakness all make individuals with PWS vulnerable to developing sleep disordered breathing [3,40]. It is not uncommon for children with PWS to sleep with an overextended neck to help to relieve upper airway obstruction [41], and both obese and non-obese subjects may exhibit severe OSA [10,42]. Hypothyroidism is a treatable condition that contributes to OSA in some patients. Studies have reported variable prevalence of hypothyroidism in PWS patients with rates ranging from 2.1% (similar to the general population) up to 32%, and the true prevalence remains unknown [8,43,44,45]. It is recommended that children be screened for hypothyroidism by 3 months of age and then on an annual basis to determine if they may require treatment [8].

#### OSA and Growth Hormone (GH) Therapy

Adenoidal and tonsillar hypertrophy are common contributors to OSA in all children. In children with PWS, treatment with GH may lead to accelerated growth of lymphoid tissues and the degree of hypertrophy may be related to insulin-like growth factor 1 (IGF-1) levels [46,47,48]. Adenotonsillar hypertrophy can be associated with OSA, and newly diagnosed OSA has been documented in a subset of PWS subjects treated with GH from as early as 6 weeks to up to 2 years after starting therapy [47,48,49,50,51]. The development of OSA during GH therapy is of particular concern due to several reports of sudden death in individuals with PWS undergoing GH therapy [41,46,52,53,54,55,56]. In PWS patients receiving GH therapy, most fatal events occurred during the first 9 months of therapy [12]. It is important to note that sudden death in PWS has also been reported in the absence of GH treatment, and that deaths occurring in both untreated patients and those on GH therapy are often associated with respiratory insufficiency or respiratory infection [12,22].

As there are important positive health effects associated with GH therapy in PWS—including improved body composition, better exercise capacity, increased physical activity, obesity prevention, and improvement in reaching certain cognitive and developmental milestones [57]—it is important to develop clear guidelines for patient monitoring and GH use to improve safety and efficacy. Among the current exclusion criteria for treatment with GH are untreated severe OSA and severe obesity, and it is recommended that individuals with PWS complete a polysomnography study prior to starting GH therapy [58,59]. Those individuals found to have OSA or other evidence of upper airway obstruction (such as chronic mouth breathing) should have an otolaryngology evaluation and, if warranted, adenotonsillectomy prior to starting GH treatment [58]. It is also recommended that GH therapy not be initiated during an acute respiratory illness [58]. Recommendations regarding repeat polysomnography during GH therapy vary. While the American Academy of Pediatrics recommends that polysomnography be repeated 6 to 10 weeks after starting GH therapy in children [60], others have recommended repeating polysomnography within 3 to 6 months after starting therapy [58] or as often as once a year during treatment [51].

Recommendations have also been proposed regarding monitoring and modulation of treatment doses after GH therapy has been initiated. One important measure is serum IGF-1 level which may increase in response to exogenous GH. High levels of IGF-1 and GH may contribute to overgrowth of lymphoid tissue and other tissue types, contributing to the development of OSA and respiratory difficulties in patients treated with GH therapy [58]. To minimize the risk of side effects, it is recommended that the IGF-1 levels be checked at least 2 times a year during GH therapy and that GH doses be adjusted so that the IGF-1 is maintained in an acceptable range and the IGF-1/IGFBP3 ratio remains similar to pretreatment levels [60,61]. The “Polysomnography Otorhinolaryngology IGF-1 (‘POI’) score” is one proposed method to systematically integrate lab values, polysomnography data, and otolaryngology evaluation (size of tonsils and adenoids) into a “respiratory risk” value to guide GH dosing both at the time of therapy initiation and during GH treatment [62].

### 4.2. Central Sleep Apnea (CSA) and Other Abnormalities in Sleep-Related Breathing

Importantly, there are age-related differences in the sleep apnea phenotypes seen in individuals with PWS. Central sleep apnea (CSA) predominates in infants and children with PWS who are less than 2 years old, while OSA is the most common finding in those older than 2 years [63]. The etiology underlying this higher prevalence of CSA in young children with PWS is unknown, but may be related to delays in maturation of the central ventilatory control centers, or to an abnormal apneic threshold which is defined as the arterial partial pressure of CO_2_ below which the drive to breathe is lost. Even after the tendency toward CSA has diminished, older subjects with PWS still exhibit abnormalities of ventilatory control during sleep, including higher arousal threshold to hypercapnia [64], blunted ventilatory response to hypercapnia [42], and poor arousal and cardiorespiratory responses to hypoxia [65]. These deficits may predispose individuals with PWS to developing sleep-related hypoventilation during adolescence and adulthood, as well as more severe oxyhemoglobin desaturations during sleep than controls matched for BMI, sex, and age [42,66].

### 4.3. Treatment Options for Sleep Disordered Breathing

The treatments options available for patients with PWS and sleep disordered breathing are similar to those available for the general population, although those interventions requiring patient cooperation and participation may be more challenging to implement due to behavioral difficulties. For children under 2 years old who exhibit CSA, oxygen titration study and supplemental oxygen during sleep are generally recommended [63,67]. For those with OSA, otolaryngology evaluation and consideration of adenotonsillectomy are recommended; however, as hypotonia and obesity also contribute to OSA, children with PWS may be more likely to have residual OSA persist after upper airway surgery [68]. It is therefore important to consider repeating polysomnography after adenotonsillectomy in children with PWS [68]. As some children with PWS have been noted to have post-operative complications including difficulty awakening from anesthesia and need for supplemental oxygen or reintubation, close post-operative observation is essential and it is important to consider post-operative hospital admission, especially for those patients with severe OSA [69]. Central apnea frequency may also increase after adenotonsillectomy and is another reason to recommend close post-operative observation [68]. Velopharyngeal dysfunction with hypernasal speech is sometimes seen in children with PWS following adenotonsillectomy and may require additional surgery such as pharyngeal flap [70].

Over time, untreated severe OSA may lead to cardiovascular complications including hypertension, cor pulmonale, and stroke [71,72,73]. For PWS patients with residual OSA persisting after airway surgery, treatment with continuous positive air pressure (CPAP) is recommended [10,74]. Weight loss may also be helpful. Traditionally this has been approached through diet and behavioral modifications [75], but more recently laparoscopic sleeve gastrectomy has also been explored as an option [76,77]. In one case, success was reported using intensive inpatient rehabilitation and interdisciplinary behavioral interventions for weight loss in a patient with life-threatening obesity [78], although the resources required for this approach make it unlikely to be a generalizable solution.

## 5. Excessive Daytime Sleepiness and REM Sleep Disturbances

In addition to long-term adverse cardiovascular and respiratory events associated with untreated OSA, sleep that is interrupted by respiratory events and arousals is unrefreshing and may cause daytime sleepiness, worsen executive function and memory, and compromise neurocognitive development [79,80,81]. In subjects with PWS, untreated OSA and excessive daytime sleepiness due to other causes have also been associated with worsening of daytime behavior, including impulsiveness [75,82]. Interestingly, some patients with PWS continue to have difficulty with hypersomnia even after OSA has been treated, and excessive sleepiness seems to be a common trait in children with PWS even when the quantity and quality of sleep appears sufficient [75,83,84]. In some individuals, hypersomnia may be due to hypothalamic dysfunction. In others, it is associated with a narcolepsy-like phenotype that includes sleep-onset REM periods and sometimes cataplexy [85,86]. Orexin-A (hypocretin-1), a neuropolypeptide important in maintaining wakefulness, is absent or found at very low levels in individuals with narcolepsy type 1 with cataplexy [87,88]. Intermediate levels of orexin-A are also seen in cerebral spinal fluid of some PWS patients with excessive daytime sleepiness [86,89]. Some success has been reported using the stimulant modafinil for PWS patients with hypersomnia [90,91]; however, it is important to ensure that hypersomnia in these patients is not secondary to severe OSA prior to starting a stimulant.

## 6. Discussion and Conclusions

Patients with Prader-Willi syndrome have altered ventilatory control and many factors predispose them toward developing sleep-related breathing disorders, including craniofacial features, obesity, hypotonia, hypothalamic dysfunction, and GH therapy-related acceleration of lymphoid tissue growth. PWS patients also have associated behavioral difficulties that may hamper effective therapeutic intervention, such as use of CPAP for persistent OSA.

Early diagnosis and treatment of OSA is important in order to prevent cardiovascular and respiratory complications, as well as detrimental effects on sleep quality, development, and daytime behaviors. Although its prevalence in PWS is not certain, hypothyroidism may contribute to OSA and yearly thyroid function testing is recommended [8]. Alternative methods of weight management, including laparoscopic sleeve gastrectomy, are being explored [76,77].

Long-term treatment with GH for children with PWS can change the natural history of body composition, motor function, and lipid profiles when therapy is begun during the preschool years [57,92]. It has also been shown to improve ventilatory control during wakefulness and some aspects of cognitive development [15,57]. Practice guidelines emphasize the importance of testing for sleep disordered breathing before and during GH therapy, as well as monitoring of IGF-1 levels to limit the risk of side effects [51,58,60]. Implementing the “POI” scoring system for GH dosing [62] could improve safety. Several of the reported fatalities in children with PWS (both with and without GH therapy) were associated with respiratory illness [12,22], thus the benefit of closer monitoring or improved airway clearance measures during acute respiratory illnesses needs to be studied systematically. Since PWS patients may not mount an adequate cortisol stress response illness [21], testing adrenocorticotropic hormone levels at baseline and during severe illness, as well as consideration of prophylactic hydrocortisone therapy during critical illness, is also recommended [60].
